# FAN-MCCD: Fast and Accurate Network for Multi-Scale Chinese Character Detection

**DOI:** 10.3390/s21217289

**Published:** 2021-11-02

**Authors:** Manar Alnaasan, Sungho Kim

**Affiliations:** Department of Electronics Engineering, Yeungnam University, 280 Daehak-ro, Gyeongsan-si 38541, Korea; manaralnaasan@gmail.com

**Keywords:** simple pipeline, multi-scale Chinese character detection, handwritten in old documents, multiscale feature network

## Abstract

Inaccurate localization due to scale-variation during character detection causes a widespread issue overconfidence in results of the document analysis community, for the most part in historical and handwritten documents. In this work, we explored the performance of a state-of-the-art network with a simple pipeline that fast and accurately predicts handwritten Chinese characters in old documents. In order to adapt to locations of characters with multi-scale more precisely, excluding pre-processing and in-between steps, we utilized a network with multi-scale feature maps. Then, across each feature map, pre-selected boxes of unalike scales and aspect ratios were employed. The last step was to prune the bounding boxes, sending them to non-maximum suppression to yield the final results. Focusing on a well-designed neural network architecture and loss function that presents well-classified examples, we found our experiments on Caoshu, Character, and Src-images datasets demonstrated that detection performance was enhanced for the detection rate (DT), the false positive per character (FPPC), and the F-score in the order of 98.84%, 0.71, and 97.64%, respectively. In comparison with SSD (single-shot detector), the detection performance of a detection rate (DT), the false positive per character (FPPC), and the F-score were 61.12%, 6.12, and 60.33%, respectively.

## 1. Introduction

**Figure 1:**
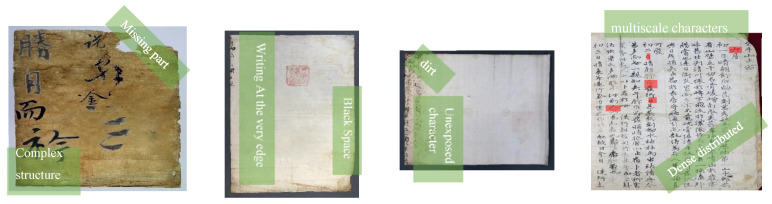
The challenges of handwritten Chinese character detection in old documents.

Text detection is a crucial assumption of an active role in the process of text understanding. In terms of Chinese characters in old documents, more challenges arise due to many difficulties such as complexity in the structure of each character, some missing parts, dense distributed characters in the image, writing at the very edge, significant multi-scale characters, and dirt that yields a background texture noise, as shown in Figure 1.

Historical documents are irreplaceable treasures, yet they remain untranslated and incomprehensible. To understand and protect these documents, we are required to convert text and graphic symbols in real documents into digital form, which basically depends on the accuracy of character detection and recognition in the document. Unlike the historical recognition task [1,2], limited research has been conducted regarding historical character detection. However, it has been reported that the detection task can be important in cases of understanding the improvement of old Chinese characters. Moreover, accurate detection of character area can help in restoring the damaged documents.

Character-level detection techniques [3,4] for historical documents highly depend on understanding of domain specific parameters and hyperparameters that are carefully tuned and are hardly able to be adapted to the new dataset.

Recently, many deep learning-based methods [5,6,7,8] have handled the detection tasks and achieved a promising performance generally for object and text detection. However, they are still far from satisfactory because they have many stages (pre- and post-processing) and it is not easy to apply them on old documents due to densely distributed characters, complexity of the character structures, and a high-level variety of character sizes.

Generally, the aforementioned proposals have achieved excellent performance. However, either traditional or deep learning ones have incorporated several stages, which is a tedious process to optimize, leading to standard performance and a long period of time to process. Furthermore, different scales of characters have not been addressed as a main problem in old documents.

**Figure 2:**
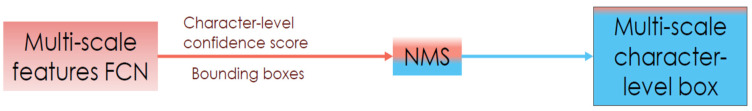
The much simpler proposed pipeline for detection the text in old documents that allows for one-stage training with no more steps that would cause wasting of time.

Scale variation across character instances is a main problem leading to an imprecise localization issue for the character detection task, which in turn is reflected in the translation result. In this paper, we propose a fast and accurate model to detect Chinese characters of various sizes in old documents. The pipeline structure is a fully convolutional network (FCN) with multi-scale feature maps that yields character-level predictions ranging from very small to very large scales directly to be sent to the non-maximum suppression that produces the final multi-scale outcomes. Figure 2 shows our low-computation pipeline that beats SSD and other methods on Chinese character benchmarks with respect to accuracy and simplicity.

The contributions of our work are organized in three parts:

- We introduce an effective and accurate multi-scale Chinese character detector that exploits different scales and aspect ratios bounding boxes over feature maps from multiple stages to directly produce character predictions and eliminate costly steps (pre- and post-processing, and in-between), which then are sent to non-maximum suppression to yield final outcomes.
- The simplicity of our end-to-end character-level pipeline stands for the effectiveness of multi-scale Chinese character predictions in challenging old documents.
- Without bells and whistles, our proposed system significantly outperforms the up-to-the-minute SSD method in terms of simplicity and accuracy on Caoshu, Character, and Src-images datasets.

## 2. Related Work

The recent approaches conducted regarding text detection have made countless efforts on multiscale text: page-level, text-line, word-level, or character-level. These methods fall into two groups. The first one is conventional bottom-up methods [9,10,11,12,13], which use either texture-based concepts that exploit a discrete cosine transform (DCT), a Fourier spectrum, or a Gabor filter to handle the text as a particular texture layout for processing, or region-based concepts that use popular methods such as stroke width transform (SWT) and stroke feature transformation (SFT) to extract candidate parts and remove no-text segments using a classifier or filter.

Although the traditional methods achieved good text-region extraction at different scales, they fell short in text detection. The second group consists of methods that are based on deep-learning top-down CNN approaches [14,15,16,17,18,19]. Inspired by the most famous techniques, the rotation region proposal networks (RRPN) proposed by Ma et al. [20] was based on Faster R-CNN [21]. The RRPN was employed for many neural networks to handle orientation text [14,20]. Moreover, Text-Boxes presented by Liao et al. [14] was based on the single-shot detector (SSD). In spite of the fact that these techniques, based on deep learning, achieved promising performance for various scales of text detection, they still suffer from low efficiency due to the localization problem, especially in old documents containing dense distributed characters with specific format, which makes it difficult to apply the aforementioned approaches appropriately enough. Pertaining to historical documents, much research has been conducted recently for analyzing such documents. In this regard, the two kinds of approaches consist of conventional [22,23,24,25] and deep learning-like methods [26,27,28,29,30,31,32] to deal with detection of text in old documents. Phan et al. [22] extracted characters depending on analyzing connected components. Liu et al. [23] described the character after being matched with reference one to determine the relation between stroke and inter-stroke. Moreover, for more effective performance, Tao et al. [24] proposed an algorithm that finds the similarity of Chinese characters using a kernel version of the discriminative locality alignment (DLA). Finally, depending on Fisher linear discriminant, Stefano et al. [25] made the performance of classification better by presenting a GA-feature selection algorithm, which successfully discriminates the samples of different classes. However, the pre-mentioned methods cannot be obtained to new datasets since they depend significantly on hyper-parameters that have different optimal values for different datasets.

On the other hand, CNN deep learning-built methods have been increasingly used for the same detection purpose. Yang et al. [26] presented a recognition guided detector (RGD) for tight and dense Chinese character detection in old documents; aiming to discover the region of character precisely using a pre-estimated region obtained from another CNN regression network (sharing parameters technique), their system achieved more accurate detection than previous conventional methods. However, it is not simple in structure due to the steps of text segmentation, proposal generation, and method obtained for bounding box creation. Further, despite the use of sharing parameters to speed up the training, it is still not perfect enough for the character detection task because it still aches from the mis-localization problem. Ahmad et al. [31] suggested a new page segmentation method that uses Siamese network to find the difference between patches; then, the extracted features were used to segment the page into main and side text regions, which means the authors handled the problem of pre-processing steps for document analysis without addressing the problem of word or character detection and recognition. In addition, expensive time was used for extracting the feature for every possible patch. To that end, page segmentation was achieved with a good result, but it was not effective in the case of using next steps for word or character level detection. The method of Dona et al. [32] focused on the problem of the scarcity of the ground truth dataset needed for most deep learning techniques. Moreover, a proposal for text (word and character level) recognition was presented; this algorithm obtained acceptable performance in terms of the character and the word error rate, although the prediction results showed some noise and did not match perfectly.

In our work, we devised a character-level FCN-based detection network that quickly and accurately detects Chinese characters in old documents at different scales. The model is optimized and learnt by end-to-end manner, and the simplicity of structure allows for the enhancement of the performance by a marked margin in comparison with SSD and other existing techniques in terms of accuracy and speed.

## 3. Methodology

**Figure 3:**
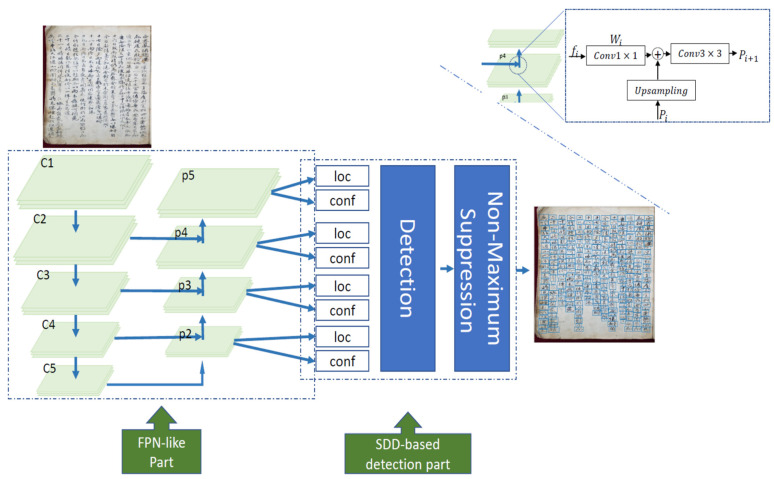
The architecture of the proposed FAN-MCCD: the FPN-like part, which is a multi-scale feature using fully convolutional network (FCN) with channels of halved-value for the purpose of obtaining characters at different sizes; the SSD-based part, which depends on pre-selected boxes using focal loss for more accurate classification during detection and to suppress the background noise problem in old documents; and the NMS for pruning these bounding boxes. The upper right corner explains the merging process using element wise addition.

Our proposed Fast and Accurate Network for Multi-scale Chinese Character Detection (FAN-MCCD), depicted in Figure 3, is an end-to-end network that detects dense and multi-scale characters existing in the image simultaneously. The first part is a feature extractor (FCN) fully convolutional network designed for such a pyramid concept; then, default boxes with different scales and aspect ratios over each feature map are used to detect large- and small-scale characters. Finally, NMS is exploited to filter the huge number of predicted boxes.

### 3.1. Proposed Feature Extractor

Scales of character areas vary enormously in old Chinese documents, leading to inaccurate localization problems, especially when coming up against the small size of characters. In such a case, early layers with high-level resolution are required to determine the presence of the small-scale characters. On the flip side, to discover the existence of the large-scale characters, we must use late layers with low-level resolution. For the sake of these prerequisites, FPN-like is exploited as the backbone network due to the multi-feature structure that can work particularly well with multi-size targets.

FPN consists of a bottom-up pathway, a top-down pathway, and lateral connections. The novelty of the suggested network stands for that the bottom-up pathway, inspired by U-Net [33], consists of a fully convolution network (FCN) with low-cost computation, which means faster to train, instead of convolutional neural network (CNN) used in the original FPN to extract features. Furthermore, we doubled the number of channels for convolutions in the down-sampling branch for more computation efficiency, instead of multiplying by 4 as in the original FPN. Here, each stage’s last residual block is used as the output of ResNet52. These outputs are annotated {*C*2, *C*3, *C*4 and *C*5} for *Conv*2, *Conv*3, *Conv*4 and *Conv*5, respectively. Like the original paper of FPN, *Conv*1 has not been included into the feature pyramid, owing to the huge memory effect. The top-down branch consists of multi-stage feature maps with up-sampling layers for better resolution. Unlike the original FPN, *P*6 feature map is not taken into account in our work due to its low resolution for the task of character detection, as will be illustrated in the upcoming experiments. As a result, the feature pyramid picked up involves {*P*2, *P*3, *P*4 and *P*5} instead of {*P*2, *P*3, *P*4, *P*5 and *P*6} for RPN.

Afterwards, skip connections are needed between down-sampling layers and the corresponding feature maps for more accurate detection and localization.

**Figure 4:**
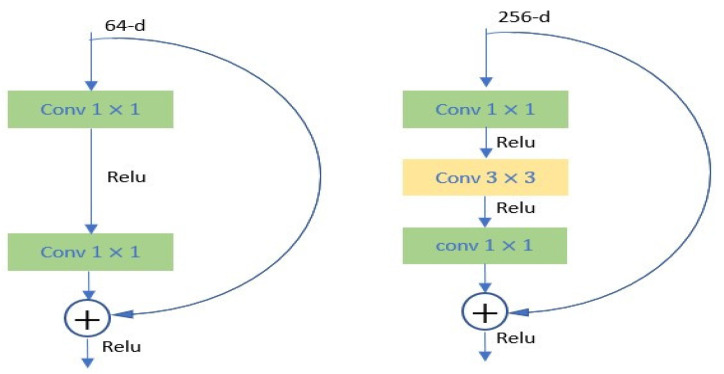
The function F of deep residual operation. Left: the block design for ResNet34. Right: the bottleneck block design for ResNet52 (thinner block).

In SSD detector, the visual geometry group (VGG) network [34] is used. However, due to the degradation problem that causes imprecision in detection with an increase in network depth, we used deep residual (ResNet) network [35] to improve the performance of the feature extractor. ResNet uses shortcut connections to make a reference to inputs and to attain plentiful deeper network. The following equation provides us with the output of ResNet:

(1) $$y=F(x,\{W_{i}\})+x$$

where *y* and *x* are the output and the input of the ResNet block, respectively, and *W_i_* is the *i*th convolutional layers parameters to be learned, whereas the action *F* + *x* is achieved using an identity shortcut with element-wise addition. Compared to ResNet34, we used ResNet52 to eliminate training time concerns since the bottle neck reduces the number of parameters and matrix multiplications, and for practical considerations, deeper and faster network of the bottleneck design. Figure 4 shows the difference between two designs.

Finally, we denoted the whole process as element-wise combination, R(x) (Figure 3, upper right corner), which represents the $\phi \left(f_{i},\;W_{i}\right)$ operation that defines the output features of ResNet52 and different 1×1 kernels, adding 1×1 kernel to reduce the number of channels to 256 for merging purpose, with the process of using 3×3 convolution alleviating the low resolution caused by up-sampling in the bottom-up branch. The following equation summarizes the feature extraction part:

(2) $$P_{i}=\{\begin{array}{c} R(\left(conv_{3\times 3}(f_{i},W_{i})\right)+P_{i+1})\;\;\;\;\;\;for\;\;\;\;i=\left\{2,3,4\right\}\\ conv_{3\times 3}\left(f_{i},W_{i}\right)\;\;\;\;\;\;\;\;\;\;\;\;\;\;\;\;\;\;\;\;\;\;\;\;for\;\;\;\;\;\;\;\;\;i=\left\{5\right\}\; \end{array}\;$$

where x is the merged map for i ϵ {2, 3, 4}, and $P_{i}$ is the output map of FPN-like network.

### 3.2. Default Boxes and IOU

Simultaneously related to each default box, a filter of 3 × 3 size is used over each feature map to estimate four bounding box coordinates, which are called offsets ∆ (*cx*, *cy*, , *h*); this estimation attempts to closely match the ground truth boxes. Moreover, the filter synchronously yields class scores for all categories (*c*1, *c*2, …, *cp*). Default boxes significantly smooth the regression task since predictions start with pre-computed priors instead of starting from scratch.

These priors are computed in such a manner that their intersection over union (IOU) ratio with respect to the ground-truth box is greater than Jaccard threshold. this strategy is considered a good starting point.

Default boxes significantly smooth the regression task since predictions start with pre-computed priors instead of starting from scratch.

These priors are computed in such a manner that their intersection over union (IOU) ratio with respect to the ground-truth box is greater than Jaccard threshold, which is considered a good starting point in order to regress closer to the original ground-truth box, and in principle, this allows our network to generalize any type of input.

**Figure 5:**
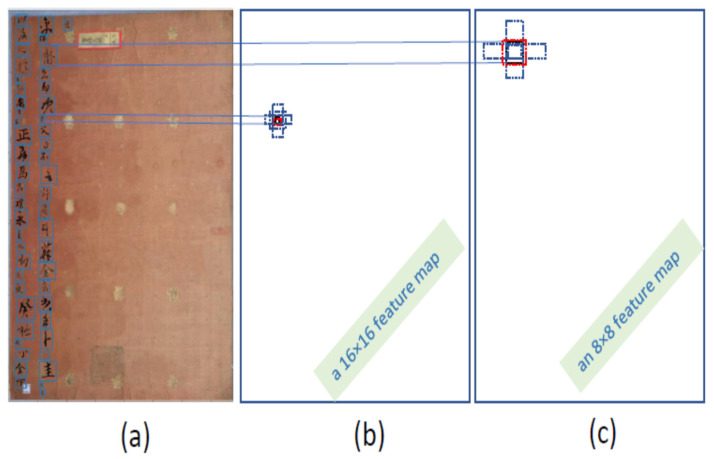
The process of multi-scale character detection: (**a**) image with ground truth boxes; (**b**) a 16 × 16 feature map with higher resolution detects a smaller character (red bounding box); (**c**) an 8 × 8 feature map with lower resolution detects a larger character (red bounding box). Over each feature map, there are a number of default boxes (dotted ones) at the given position, and the red box means that it is the proper one for character size.

Our proposed FAN-MCCD precisely detects multi-scale characters using a prediction technique that computes offsets and confidence scores for multi-resolution feature maps. The feature maps with low-context information detect large-scale characters, while the feature maps with high-context information detect small-scale characters. As a case in point, the 16 × 16 feature map with high resolution in Figure 5b detects the characters with a smaller size. On the contrary, the 8 × 8 feature map with low resolution in Figure 5c detects the characters with a larger size.

### 3.3. Proposed Multi-Box Loss

FAN-MCCD uses the multi-box regression technique; this technique combines two critical components of loss functions represented by Confidence loss and location loss, as seen in Equation (2).

(3) L(x ,c,l,g)= 1/N (L_conf (x,c)+αL_loc (x,l,g)) 

*N* indicates how many default boxes are matched, and the hyper-parameter *α* supervises the trade-off between confidence and location losses. In our experiments, *α* is set to 1.

Location loss: Given that the *L2-Norm* is stable and more precise, we exploited it in our experiment instead of the *L1-Norm*.

Equation (3) is used to match the predicted box *l* with the ground truth box *g* in terms of (*cx*, *cy*, *h*, *w*) parameters, which are center coordinates for the first two arguments and the height and width of default box offsets *d*, respectively.

(4) $$L_{loc}=\overset{N}{\underset{i\in Pos}{\sum }}\underset{m\in \left\{cx,cy,w,h\right\}}{\sum }x_{ij}^{k}\;L2-Norm{\left({\hat{l}}_{i}^{m}-{\hat{g}}_{j}^{m}\right)}^{2}$$

$${\hat{g}}_{j}^{cx}=\frac{\left(g_{j}^{cx}-d_{i}^{cx}\right)}{d_{i}^{w}}\;\;\;\;\;\;{\hat{g}}_{j}^{cy}=\frac{\left(g_{j}^{cy}-d_{i}^{cy}\right)}{d_{i}^{h}}\;\;\;\;\;\;{\hat{g}}_{j}^{w}=log\left(\frac{g_{j}^{w}}{d_{i}^{w}}\right)\;\;\;\;\;\;\;{\hat{g}}_{j}^{h}=log\left(\frac{g_{j}^{h}}{d_{j}^{h}}\right)\;$$

Classification loss: Object classification is performed by our proposed network. In this regard, for each class and predicted bounding box, FAN-MCCD computes a group of *c*-sets. Thus, to handle the class imbalance issue emerged by the background noise due to the dirt and other reasons of long-term storage of documents, the confidence loss applied is a focal loss in place of a cross entropy loss over multiple classes (Equation (4)).

(5) $$L_{conf}=-\overset{N}{\underset{i\in Pos}{\sum }}{\left(1-{\hat{c}}_{i}^{p}\right)}^{\gamma }\;x_{ij}^{p}\left({\hat{c}}_{i}^{p}\right)-\underset{i\epsilon Neg}{\sum }{\left(1-{\hat{c}}_{i}^{0}\right)}^{\gamma }\log\left({\hat{c}}_{i}^{0}\right)\;\;\;\;\;in\;here\;{\hat{c}}_{i}^{p}softmax=\frac{exp\;{\left(1-c_{i}^{p}\right)}^{\gamma }}{\sum_{p}exp{\left(1-c_{i}^{p}\right)}^{\gamma }}$$

$x_{ij}^{p}$ is a matching matrix of class *p* between the *i*-th bounding-box and *j*-th ground-truth-box. In the case of matched boxes, the matrix gives a value of 1, but 0 otherwise.

### 3.4. Matching Technique

The most important question that comes to mind is “What is the strategy of matching boxes?”.

The logic revolving around the creation of a default box is somewhat complicated but is still within our grasp.

For a multi-box task, pre-computed anchors are generated by researchers (or they are called priors in the FasterRCNN).

In fact, those anchors are created in such a way that the intersection over union is greater than 0.5 (IOU > 0.5). Unlike the Faster-RCNN, our FAN-MCCD matches the ground truth boxes with the default boxes and selects the default box with highest IOU score. Then, to simplify training, the proposed network keeps the default boxes matched with ground truth and IOU that is greater than 0.5 (as in SSD).

### 3.5. Online Hard Example Mining (OHEM)

After the aforementioned matching technique, the interpreted unmatched boxes (background) as negatives consist of a large amount of low IOU in the training set, which in turn produces a class-imbalance between the character and background examples. Instead of using each negative prediction, we chose to pick the best ones, and to do that, we set a ratio of three negative backgrounds to one positive character.

### 3.6. Augmentation Sorts

To adapt to multi-scale characters, we used data augmentation. To that end, our dataset was augmented using rotation of background, boldness and size of character, random crop, horizontal flips, and photogenic distortions of random brightness and contrast. For the rotation of background, boldness, and size of character, we deployed synthesized images for augmentation purposes, where background images were rotated randomly and characters were fetched to be synthesized, and inspired by [36], scale augmentation with randomly sized characters was used keeping the aspect ratio fixed. In addition, characters were written to the corresponding position with a random value of boldness.

These augmentation methods improve the detection performance for low resolution input.

### 3.7. Training

FAN-MCCD is an end-to-end network. For more efficient and easier learning, focal loss was used. Moreover, FCN with doubled-channels as a feature extractor was employed to obtain a light-weighted network that directly targets the text detection goal.

## 4. Experiment

In order to prove FAN_MCCD detection performance, we employed an experiment to show a comparison with other approaches in terms of some protocols and in terms of dataset visualization. Starting with implementation details of our model, we simply illustrated that the network reached the optimal values during training shift. Supplementary Materials which provide a video of detection performance using prediction bounding boxes (in blue) and ground truth ones (in red) to show the accuracy of our model is obtained.

### 4.1. Implementation Details

The model was implemented on PyTorch. The amount of data regarding the specific issue was not large enough to train the network from scratch. In such a scenario, ResNet52 pretrained with ImageNet dataset was used for the feature extractor part. The training process was 30 epochs long, with a batch size of 1 to deal with a higher resolution of input image since larger size means better detection staying within GPU memory. Adam optimizer was employed with a detection rate of 0.0001. Additionally, for a more robust network, we obtained data augmentation. First, random background images were rotated by 90 degrees. Next, the boldness and the size of each character were set to random. Then, arbitrarily cropped and horizontally flipped images were used. Finally, arbitrary brightness and contrast were exploited. The merged dataset was applied for generalization purpose, as is depicted in the following sections.

As illustrated in Section 3.5, OHEM was obtained to improve the performance. For each image, we increased the negative-to-positive ratio to 3:1.

At test time, the large number of bounding boxes generated during forward pass were sent to be diminished, applying NMS to obtain the ultimate detection results.

### 4.2. Benchmark Datasets

We evaluate our proposed FAN-MCCD on three kinds of old document datasets and on background-only images. These data were collected by Kyungpook National University KNU (available on: http://dila.co.kr/index.php, accessed on 25 October 2021), and they were scanned or photographed documents of separately handwritten characters in the Chinese language.

Caoshu dataset consists of 1000 images for training and 500 images for testing. These images are documents scanned with dense distributed characters.

Character dataset contains 300 images for training and 200 images for testing. Most of these images’ spaces are empty, with a few vertical lines of characters.

Src-images dataset combines 500 images for training and 200 images for testing. They are character-cropped images of very large sizes.

**Figure 6:**
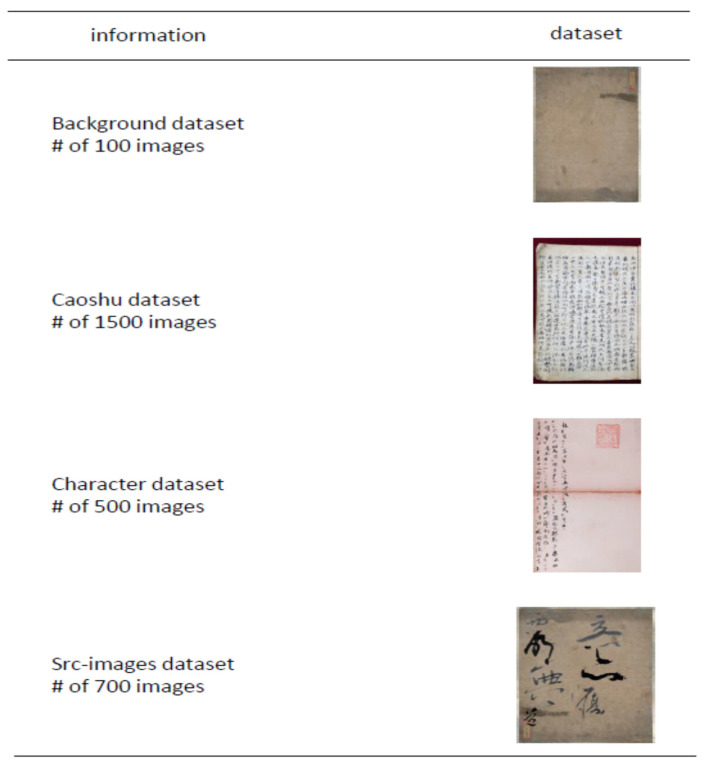
Samples of the KNU dataset used in our experiments with some information that provides the name and the number of images-obtained for each group.

Background-only images for augmentation purposes, including 100 no-character images that are empty images with background only. As depicted in Figure 6.

### 4.3. Comparison with State-of-the-Art SSD and Other Algorithms

**Figure 7:**
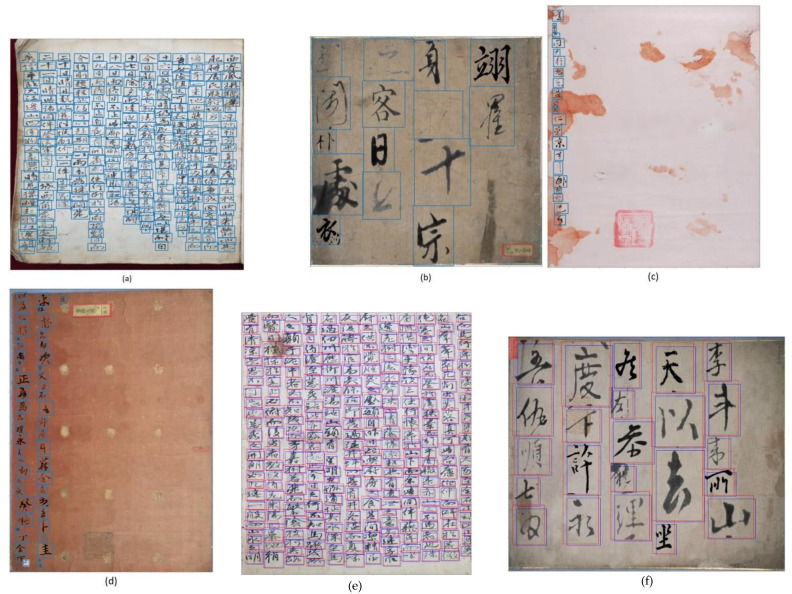
The results of the proposed FAN-MCCD that effectively adapts all different scales of characters in all different kind of KNU datasets. (**a**) The detection result on Caoshu dataset with dense distribution and different sized characters. (**b**) The detection result on Src-images dataset with very large multi-scale characters. (**c**,**d**) The result on Character dataset with a small number of various scales of characters. (**e**,**f**) The results with predicted bounding boxes (blue) overlaid with ground truth bounding boxes (red).

**Figure 8:**
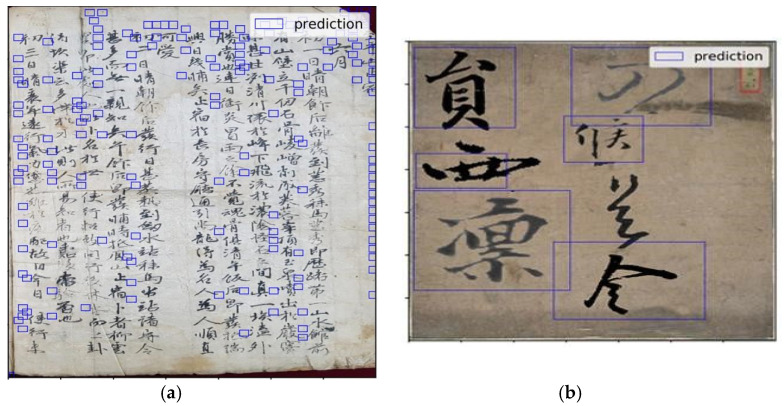
The result of SSD on KNU datasets. (**a**) The result on Caoshu dataset, clearly showing that there is a serious problem with detection emerged from inaccurate localization. (**b**) The result on Src-images with good detection.

This section presents an evaluation of our proposed method with SDD and other algorithms for a merged dataset with augmented characters using different IOU ratios. As shown in Table 1 and Table 2, our proposed method achieved better performance than the state-of-the-art SSD and other algorithms for all IOU ratios selected and in terms of detection rate, false positive per character and F-score. As a result, we found that the proposed FAN-MCCD adapts to multi-scale characters better than SSD and other algorithms due to its structure, which has different feature maps of different dimensions, and all these feature maps, starting from the bottom (ignoring the first large one only) until the end, were used for detection purposes. As depicted in Figure 7, whether the size of the character was too-small or too-large, our model was able to effectively detect handwritten characters with multiple scales in old documents. This is what makes our proposed algorithm the best among the other algorithms used for evaluation. In addition, to show how the proposed model is accurate, we visualized the predicted boxes overlaid with ground truth ones. Figure 8 shows results on Caoshu and Src-images for SSD algorithm. As is shown, there was a serious problem related to character localization for the Caoshu dataset. Although SSD was constructed for multi-scale purposes, it could still impose inaccurate localization for small characters.

### 4.4. Effectiveness of Different Layers

Unlike the FPN network, our model uses {*P*_2_ *P*_3_ *P*_4_ and *P*_5_} and excludes the {*P*_6_} feature map. For the reason that FAN-MCCD is employed for the character-level detection task, some layers in the feature pyramid can be dumped, considering that the extraction effectiveness of some layers is likely not clear.

Table 3 shows the layer change effects, and it is obvious that removing {*P*_5_} affected the detection results significantly. Using another logic of removing {*P*_2_} showed that the detection rate remarkably declined, whereas removing {*P*_6_} did not affect detection that much since the scale of {*P*_6_} was too small. The above-mentioned study appeared to show that {*P*_5_}, which is produced by {*P*_4_} upscaling, is the main part since it provides information about the character region more precisely and has stronger semantic values.

### 4.5. Effectiveness of the Positive Anchor Number

In our FAN-MCCD, default boxes were precisely pre-picked anchors that remarkably affected the training process. Hence, the positive number of boxes was the main problem; if that number decreased significantly during the training phase, our network would yield an overfitting problem. Table 4 illustrates how the value selected for IOU affected the number of positive anchors importantly. Here, increasing IOU threshold led to decreasing the positive number of default boxes, which in turn failed to match ground truths, and this issue severely affected the training predictor at high value of IOU, which was 0.8 in our case with only three positive boxes left. In that case, we did not have adequate information for efficacious training.

## 5. Conclusions

This work presents a handwritten multi-scale Chinese character detector with a simple pipeline that provides character-level predictions in old documents. We employed a network with feature maps from different stages to match characters of different sizes. This network is a single stage by optimizing a multi-task loss. FAN-MCCD inherits the advantages of multi-level detection with focal loss to achieve the goal of fast, accurate, and well-classified characters. The experimentally evaluated model (FAN_MCCD) on old document benchmarks confirms that the presented algorithm significantly outperforms SSD detector and other previous methods in terms of accuracy and efficiency. On the other hand, the proposed method is not suitable for multi-lingual recursive text that is written in old documents. For future directions, we plan to include the improvement of our system to accommodate this. Moreover, we plan to integrate the system with a text recognition task.

## Figures and Tables

**Table 1:** Comparison between FAN_MCCD and SSD with different values of IOUs in terms of detection rate (DT), false positive per character (FPPC), and F-score.

Approach	IOU	DT	FPPC	F-Score
CCB_SSD [37]	0.5	61.22%	5.20	60.41%
	0.6	61.12%	6.12	60.33%
	0.7	60.32%	8.11	59.20%
FPN_MCCD (Ours)	0.5	98.84%	0.71	98.64%
	0.6	98.45%	0.72	97.55%
	0.7	97.45%	0.75	96.33%
RGD	0.5	98.32%	5.00	97.60%
	0.6	80.12%	6.72	96.82%
	0.7	97.30%	7.69	94.82%
YOLO [38]	-	-	-	-

**Table 2:** Comparison between FAN-MCCD, SSD, and other algorithms with different values of IOUs in terms of detection rate (DT), false positive per character (FPPC), and F-score.

Approach	IOU	DT	FPPC	F-Score
SSD	0.5	61.22%	5.20	60.41%
	0.6	61.12%	6.12	60.33%
	0.7	60.32%	8.11	59.20%
FPN_MCCD (Ours)	0.5	98.84%	0.71	97.64%
	0.6	98.45%	0.72	97.55%
	0.7	97.45%	0.75	96.33%

**Table 3:** Detection rate (DT) with different feature maps.

Layers				
Dataset	{*P*_2_ *P*_3_ *P*_4_}	{*P*_3_ *P*_4_ *P*_5_}	{*P*_2_ *P*_3_ *P*_4_ *P*_5_}	{*P*_2_ *P*_3_ *P*_4_ *P*_5_ *P*_6_}
Caoshu	97.10%	96.65%	98.13%	98.10%
Character	96.00%	96.99%	98.79%	98.70%
Src-images	98.72%	97.53%	98.80%	98.83%
Merged dataset	97.80%	97.69%	98.84%	98.82%

**Table 4:** Number of anchors matched per character with different IOU thresholds.

IOU Threshold	SSD Detector	FAN-MCCD (Ours)
0.5	20.21	20.21
0.6	6.07	5.05
0.7	3.01	4.06
0.8	2.62	3.01

## Data Availability

The Chinese figures used in the manuscript are collected and cre-ated by cooperation between Yeungnam university team and Kyungpook national university team. The permission by the other team has been obtained and no need for copyright since it is our own dataset. The database is available online (http://dila.co.kr/index.php, accessed on 28 October 2021).

## Supplementary Materials

The following are available online at https://www.mdpi.com/article/10.3390/s21217289/s1, Video S1: Chinese character detection with FAN-MCCD end-to-end network. A video of detection results using prediction and ground truth boxes for a more satisfied comparison.
